# MELK Inhibition Effectively Suppresses Growth of Glioblastoma and Cancer Stem-Like Cells by Blocking AKT and FOXM1 Pathways

**DOI:** 10.3389/fonc.2020.608082

**Published:** 2021-01-14

**Authors:** Xu Zhang, Jie Wang, Yifeng Wang, Guanzheng Liu, Huan Li, Jiefeng Yu, Runqiu Wu, Jun Liang, Rutong Yu, Xuejiao Liu

**Affiliations:** ^1^ Institute of Nervous System Diseases, Xuzhou Medical University, Xuzhou, China; ^2^ Department of Neurosurgery, Affiliated Hospital of Xuzhou Medical University, Xuzhou, China; ^3^ The Graduate School, Nanjing Medical University, Nanjing, China; ^4^ Department of Neurosurgery, The Second Affiliated Hospital of Xuzhou Medical University, Xuzhou, China

**Keywords:** glioblastoma multiforme, glioblastoma stem-like cells, maternal embryonic leucine-zipper kinase, OTSSP167, targeted therapy

## Abstract

Glioblastoma multiforme (GBM) is a devastating disease yet no effective drug treatment has been established to date. Glioblastoma stem-like cells (GSCs) are insensitive to treatment and may be one of the reasons for the relapse of GBM. Maternal embryonic leucine zipper kinase gene (*MELK*) plays an important role in the malignant proliferation and the maintenance of GSC stemness properties of GBM. However, the therapeutic effect of targeted inhibition of *MELK* on GBM remains unclear. This study analyzed the effect of a MELK oral inhibitor, OTSSP167, on GBM proliferation and the maintenance of GSC stemness. OTSSP167 significantly inhibited cell proliferation, colony formation, invasion, and migration of GBM. OTSSP167 treatment reduced the expression of cell cycle G2/M phase-related proteins, Cyclin B1 and Cdc2, while up-regulation the expression of p21 and subsequently induced cell cycle arrest at the G2/M phase. OTSSP167 effectively prolonged the survival of tumor-bearing mice and inhibited tumor cell growth in *in vivo* mouse models. It also reduced protein kinase B (AKT) phosphorylation levels by OTSSP167 treatment, thereby disrupting the proliferation and invasion of GBM cells. Furthermore, OTSSP167 inhibited the proliferation, neurosphere formation and self-renewal capacity of GSCs by reducing forkhead box M1 (FOXM1) phosphorylation and transcriptional activity. Interestingly, the inhibitory effect of OTSSP167 on the proliferation of GSCs was 4-fold more effective than GBM cells. In conclusion, MELK inhibition suppresses the growth of GBM and GSCs by double-blocking AKT and FOXM1 signals. Targeted inhibition of MELK may thus be potentially used as a novel treatment for GBM.

## Introduction

Glioblastoma multiforme (GBM) is the most common brain cancer in adults and is associated with a high mortality rate and poor prognosis (1, 2). Surgery is the major treatment for GBM, which is also combined with postoperative radiotherapy and chemotherapy. However, the median overall survival of GBM patients is less than 15 months, and the 5-year survival rate is less than 5% (3, 4). No effective targeted drug for GBM treatment is currently available, and thus the identification of new therapeutic strategies, particularly targeted therapy such as small-molecule drugs, is imperative.

Glioblastoma stem-like cells (GSCs), also known as brain tumor stem cells or promoter cells, are a subpopulation of GBM (5). GSCs have high carcinogenic potential, self-renewal ability, and multidirectional differentiation ability. It is considered to be the driving force for supporting GBM development, invasion, and anti-treatment, as well malignant recurrence (6). Because GSC has a variety of drug-resistant molecules, it is not sensitive to external physical and chemical factors that can kill tumor cells (7). Numerous studies have shown that inhibiting the self-renewal capacity and differentiation of GSC slows cancer progression (8, 9). Therefore, the eradication or permanent blockage of GSCs helps eliminate the formation and recurrence of GBM, and targeting GSC is essential.

Maternal embryonic leucine-zipper kinase (MELK) is a member of the Snf1/AMP-activated protein kinase (AMPK) family and is involved in cell cycle regulation, cell proliferation, apoptosis, and tumor formation (10, 11). Inhibition of MELK significantly increases the sensitivity of various tumor models to radiotherapy and chemotherapy (12–14). MELK also plays an important role in tumor resistance and DNA repair (13, 15, 16). It is also an important carcinogen for the pathogenesis of GBM (17). MELK expression is negatively correlated with GBM survival rate (18). MELK regulates GBM malignant proliferation and progression (18). In addition, high MELK expression is closely related to the maintenance of stemness properties of GSCs. MELK promotes neurosphere formation and differentiation of GSCs *via* the MELK/cellular Jun (c-JUN) or MELK/forkhead box M1 (FOXM1) pathway (19, 20). Small interfering RNA (siRNA)-mediated degradation of MELK induces apoptosis of GSCs *in vitro*. However, it has a weaker inhibitory effect on normal neural progenitor cells (NPCs) (21). Thus, MELK is a key regulator of GSC survival, which increases the potential use of MELK as a therapeutic target.

The inhibition/inactivation of MELK may be a potential treatment strategy for GBM. OTSSP167 is an orally available MELK inhibitor that is current in phase I/II clinical trials for various tumors (22). MELK is stabilized by autophosphorylation. OTSSP167 has been described to inhibit MELK activation by blocking autophosphorylation of MELK, thus resulting in the degradation and loss of MELK protein thereby inhibiting the phosphorylation of MELK substrate molecules (12, 23). Nano-grade OTSSP167 effectively inhibits the proliferation of many tumors that exhibit high MELK expression; however, it has no inhibitory effect on tumors or normal cells that have low MELK expression (24). To date, the potential impact of OTSSP167 on GBM proliferation and GSC stemness maintenance remains unclear.

This study investigated the effect of a MELK inhibitor, OTSSP167, on GBM proliferation *in vivo* and *in vitro* as well as on GSC stemness. We also analyzed the potential mechanism of OTSSP167 in GBM treatment.

## Methods and Materials

### Cell Lines and Reagents

Human GBM cells lines (U87, U251, A172, T98G, LN229 and LN18) used in this study were cultured and maintained in Dulbecco’s modified Eagle’s medium (DMEM) supplemented with 10% fetal bovine serum (FBS). These cell lines were grown in a humidified incubator containing 5% CO_2_ at 37°C. MELK (cat.no.2274s), AKT (cat.no.4691s), p-AKT(Ser473, cat.no.4058s), p-mTOR (Ser2448, cat.no.5536s), p-S6 (Thr389, cat.no.9206s), p21 (cat.no.2947s), Cyclin B1 (cat.no.12231s), Cdc2 (cat.no.77055s), FOXM1 (cat.no.20459s), p-FOXM1(Ser35, cat.no.14170s) and β-actin (cat.no.8457S) primary antibodies were purchased from Cell Signaling Technology (CST, Beverly, MA, USA). Antibody for Ki-67 (Cat.PA5-16446) was purchased from Thermo Fisher (Waltham, MA, USA). MELK inhibitor OTSSP167 and AKT inhibitor MK-2206 were purchased from Sellect Chemicals (Houston, TX, USA). OTSSP167 and MK-2206 were dissolved in DMSO to create a 10 mmol/L solution, which was diluted to different concentrations of DMEM medium before use.

### Culture of GSCs

GSC1 and GSC2 were derived from patients who were diagnosed with glioblastoma. These two GSC lines were cultured in neurobasal medium containing basic fibroblast growth factor, epidermal growth factor, B27 supplement, harpin, L-glutamine, and N2 supplement to form a neurosphere culture that is enriched with GSCs. A third volume of fresh medium was added every three days, and neurospheres were dissociated using a NeuroCult Chemical Dissociation Kit (StemCells Technologies, Vancouver, BC, Canada) for cell passage according to manufacturer’s protocol.

### Cell Counting Kit (CCK)-8 Assay

Cell viability was examined using a Cell Counting Kit-8 (Dojindo, Kumamoto, Japan) as previously described (25). The GBM cells were seeded into 96-well plates with 3,000 cells per well and cultured overnight, followed by the addition of different concentrations of OTSSP167. After 72 h of treatment, 10 μL of CCK-8 solution were added to each well, followed by incubation for 2 h and measuring the absorbance (optical density, OD) at a wavelength of 450 nm. Three independent experiments were conducted with each experiment having three replicate wells, and the background reading of media was subtracted from each well for result standardization.

### EdU Incorporation Assays

The Cell-Light EdU Cell Proliferation Detection Kit (Ruibo Biotech, Guangzhou, China) was used for the detection of cell proliferation. The human glioblastoma cell lines, U87 and LN229, were seeded into 96-well plates. After overnight culture, the adhered cells were treated with 0–200 nM OTSSP167. After 24 h, the cells were added and continuously incubated with 50 μM 5-ethynyl-2’-deoxyuridine (EdU) for 4 h. Subsequently, the cells were fixed with 4% paraformaldehyde solution for 15 min and treated with 0.5% Triton X-100 for 20 min, followed by incubating with 1× Apollo^®^ reaction cocktail in the dark for 30 min before DPAI staining for 20 min. After washing thrice with phosphate-buffered saline (PBS), the cell images were taken under a fluorescent inverted microscope. This experiment was conducted three times.

### Colony Formation Assay

The U87 and LN229 cells were seeded into a 6-well plate (400 cells/well), with 3 replicate wells per group. After cell adhesion, the cells in the experimental group were treated with 0–200 nM OTSSP167, and the cells in the control group were treated with DMSO. After 12 h, cell lines were further incubated with fresh DMEM medium containing 10% FBS for approximately 14 days, until colony formation stopped (judged by naked eye). The cells were washed with PBS and fixed in 4% paraformaldehyde solution. After staining with crystal violet working solution and rinsing the staining solution, the colonies were observed, photographed, and counted.

### Cell Cycle Analysis

We examined the effect of OTSSP167 treatment on cell cycle distribution by flow cytometry as previously described (26). The U87 and LN229 cell lines were treated with OTSSP167, followed by cycle analysis 24 h later. Propidium iodide (PI) staining was used to analyze cell cycle distribution. The cells were collected after staining and centrifuged at 1,000 rpm for 5 min at 4°C. Subsequently, the cells were fixed in 3 mL of 70% ice-cold methanol overnight. The fixed cells were washed twice with PBS and stained with PI solution containing RNase A for 30 min. Finally, the stained cells were detected by flow cytometry, and the cell cycle distribution was analyzed using a flow cytometry software (Becton-Dickinson, Franklin Lakes, NJ, USA).

### *In Vitro* Cell Migration and Invasion Assays

Cell invasion and migration assays were performed using a Transwell system. The U87 and LN229 cells were re-suspended in serum-free DMEM medium containing various concentrations of OTSSP167 or DMSO. The cell suspensions were seeded into the upper layer of the Transwell chamber at a density of 10,000 cells per well, and the lower layer of the Transwell chamber contained DMEM medium supplemented with 10% FBS. For the cell invasion assay, diluted Matrigel in cold distilled water was applied to polycarbonate membrane filters with an 8-μm pore size. However, the migration assay did not require the addition of Matrigel. After 24 h of incubation, the non-invasive or non-migrating cells were wiped off with a cotton swab, and the cells that invaded or migrated to the lower layer of the Transwell system were fixed with 4% methanol for 20 min, followed by staining with 0.3% crystal violet solution for 30 min and observing the cells under a light microscope before imaging and counting.

### Western Blotting

The U87 and LN229 cells were treated with different concentrations of OTSSP167 separately. After 24 h, the cells were collected for total protein extraction, which was quantified by the Bradford protein assay before western blotting. The specific methods were as described previously (27, 28). Briefly, 50 μg of total protein were separated by sodium dodecyl sulfate-polyacrylamide gel electrophoresis (SDS-PAGE), and the protein gels were transferred onto polyvinylidene fluoride (PVDF) membranes, which were subsequently blocked with 5% skim milk at room temperature for an hour before incubating with specific primary antibodies at 4°C overnight. The next day, the membranes were incubated with the corresponding secondary antibodies before developing using an enhanced chemiluminescence (ECL) reagent. The relative protein expression of Cyclin B1 (dilution 1:1,000), Cdc2 (dilution 1:1,000), MELK (dilution 1:500), p21 (dilution 1:1,000), AKT (dilution 1:1,000), p-AKT(Ser473) (dilution 1:1,000), phosphorylated-mammalian target of rapamycin (p-mTOR(Ser2448)) (dilution 1:800), and p-S6(Thr389) (dilution 1:750) were analyzed using β-actin (dilution 1:2,000) as loading control.

### *In Vitro* Cell Viability and Neurosphere Formation Assays

The GSC1 and GSC2 cells were seeded into 96-well plates at a density of 1,000 cells per well and treated with the indicated concentrations of OTSSP167 or DMSO. The CellTiter-Glo luminescent cell viability kit (Promega, Madison, WI, USA) was used to assess cell viability on days 0, 3, 6, 9, and 12. For the neurophere formation assay, the GSC1 and GSC2 cells were seeded into 96-well plates at a density of 1,000 cells per well. The cells were cultured in neurobasal medium containing various concentrations of OTSSP167 or DMSO. After 10–14 days, the neurophere formation were observed under a light microscope. Neurospheres containing more than 50 cells were scored. The numbers of neurospheres in each well were calculated.

### *In Vitro* Limiting Dilution Assay

Cells were dissociated into single cells, and then plated in 96-well plates at a density of 1, 5, 10, 20, 40, or 80 cells per well, with 10 replicates for each cell number. After 7 days, the presence of tumorspheres in each well was determined. Extreme limiting dilution assays was analyzed using online software (http://bioinf.wehi.edu.au/software/elda/) (29).

### Plasmid Transfection and Luciferase Reporter Assay

FOXM1 overexpression plasmid (p3×Flag-FOXM1) was transfected into the GSC1 cells with Lipofectamine™ 2000 reagent (Invitrogen, Carlsbad, CA, USA). After transfection for 48 h, the cells were treated with 0.1% DMSO or 25 nM OTSSP167 for 24 h. The cells were harvested for western blot analysis and *in vitro* cell viability assay.

Transcriptional activity of FOXM1 was detected as our previous report (30). The Lipofectamine™ 2000 was used to transfect 6× FOXM1-luc and *Renilla* luciferase reporter vector into the GSC1 cells. After 48 h, 0.1% DMSO or different concentrations of OTSSP167 were added to the transfected cells and further incubated for 24 h. The luciferase reporter assay was performed using the Dual Luciferase Assay kit (Promega, Madison, WI, USA). The activity of sea cucumber luciferin was used as control for the transfection and expression efficiency of the experiment. All samples were repeated in triplicate.

### *In Vivo* Studies

All animal protocols were approved by the Ethics Committee of the Xuzhou Medical University (Jiangsu Province, China). Fifty-seven male athymic BALB/c nude mice aged 5–6 weeks were purchased from Beijing Vital River Experimental Animal Technology Co. Ltd., China. This study used a subcutaneous animal model and orthotopic transplantation tumor model to evaluate the therapeutic effect of OTSSP167 on GBM treatment. For the subcutaneous tumor model, the U87 cells (5 × 10^5^) were inoculated on the right lateral flank of nude mice. Once the tumor grew to a volume of approximately 50 to 100 mm^3^, the nude mice were randomly divided into 3 groups: the control group, a 5 mg/kg OTSSP167-treatment group, and a 10 mg/kg OTSSP167-treatment group. The animals in the OTSSP167-treatment groups were intraperitoneally injected with the corresponding concentrations of OTSSP167 twice a week for 4 weeks, and their tumor size was measured with calipers every 3 days. The volume of the subcutaneous tumors was calculated as follows: Tumor volume = (Length × Width^2^)/2 (assuming a prolate shape).

For the intracranial tumor model, the U87 cells (5 × 10^5^ cells per mouse) or GSC1 cells (5 × 10^4^ cells per mouse) were respectively injected *in situ* into the right striatum of nude mice using a small animal stereotaxic instrument as previously described (31). Five days after the tumor cells were inoculated, the nude mice bearing tumor cells were randomly divided into 3 groups (n = 15 mice per group). The mice were treated with OTSSP167 (5 μL of 1 μM or 2 μM OTSSP167 in 1% DMSO in PBS per mouse) or vehicle control by intratumoral injection once a week for 4 weeks. After 30 days, 5 mice were randomly selected from each group and euthanized, followed by brain perfusion to assess brain tumor size. The remaining 10 mice in each group were used for survival analysis.

### Hematoxylin-Eosin (HE) Staining and Immunohistochemistry (IHC)

The whole brains of mice in the control and treatment groups were fixed in 4% paraformaldehyde solution overnight, followed by paraffin-embedding and tissue sectioning at a thickness of 5 μm. The brain sections were placed on glass slides and dried in an oven. For HE staining, the brain sections were deparaffinized in xylene solution, hydrated across an ethanol gradient, and then rinsed with tap water. The brain sections were then stained with HE staining solutions in sequence for 5 min, followed by dehydration and mounting with neutral gum. Brain morphology was then assessed by light microscopy and imaged.

For IHC, the brain sections were heat retrieved in a microwave oven at 60°C for 30 min, followed by deparaffinizing in xylene solution for 15 min thrice and hydrating across a decreasing ethanol gradient (100, 85, and 75%) for 5 min each. The brain sections were incubated with citric acid antigen repair buffer, further washed with PBS thrice, and quenched in 3% hydrogen peroxide solution in the dark for 25 min. The brain sections were then blocked with bovine serum albumin (BSA) for 30 min followed by incubation with the corresponding primary antibodies individually (anti-MELK (dilution 1:100), anti-p-AKT(Ser473) (dilution 1:200), and anti-ki67 (dilution 1:150) primary antibodies) at 4°C overnight. Subsequently, the brain sections were incubated with the corresponding secondary antibodies (dilution 1:200) at room temperature for 50 min before developing with 3,3′-diaminobenzidine (DAB) solution. The brain sections were subjected to nuclear counterstaining with a hematoxylin solution for 3 min, followed by routine dehydration, mounting, and observation and imaging under a light microscope.

### Statistical Analysis

Each experiment was independently repeated for at least three times. The figures show representative images of our results of repeated experiments. The experimental results were statistically analyzed using GraphPad Prism 6.0 (San Diego, CA, USA). The data were presented as the means ± SEM. Comparison between two samples was performed using an independent sample *t*-test. The Kaplan-Meier method was used for survival analysis of the mice. The log-rank test was used to compare survival time between the two study groups. *P <*0.05 was considered statistically significant.

## Results

### OTSSP167 Significantly Inhibits GBM Cell Proliferation

**Figure 1:**
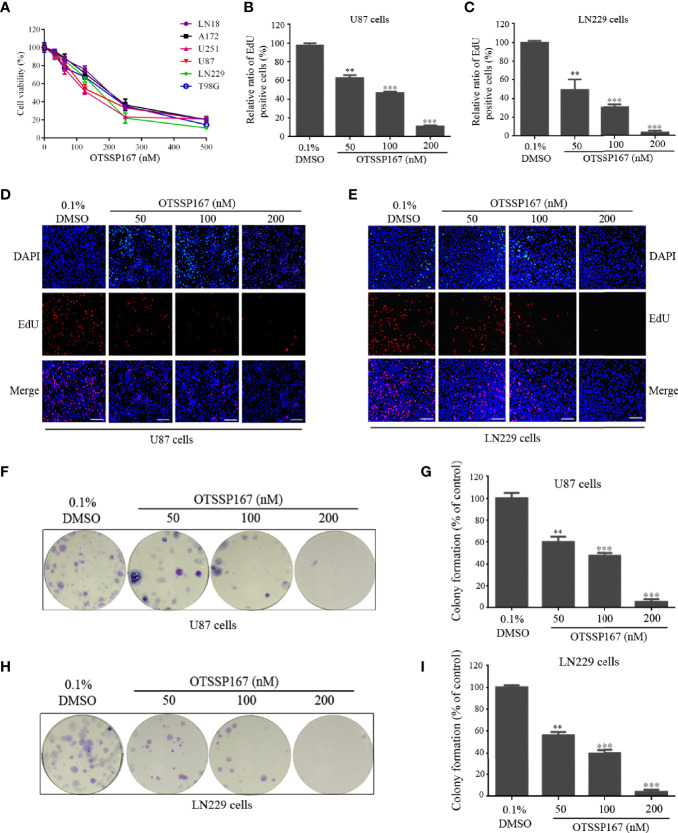
OTSSP167 inhibits GBM cell proliferation and colony formation. **(A)** CCK-8 viability analysis of cells treated with six OTSSP167 concentrations, including 0 nM, 31.25 nM, 62.5 nM, 125 nM, 250 nM, and 500 nM for 72 h. **(B–E)** The U87 and LN229 cells were treated with indicated concentrations of OTSSP167 for 24 h, and the EdU assay was performed to assess cell proliferation. Panels **(B, C)** show the results of the quantitative analysis of the EdU test; panels **(D, E)** show the representative images of EdU analysis after OTSSP167 treatment of the U87 and LN229 cells. **(F–I)** OTSSP167 inhibits colony formation in U87 and LN229 cells in a dose-dependent manner. Quantitative analysis of the results of the colony formation experiment was performed. All the Data are presented as means ± SEM. **P < 0.01, ***P < 0.001 compared with the 0.1% DMSO treated group.

To evaluate the effect of MELK inhibitor, OTSSP167 on GBM cell growth, a CCK-8 assay was performed to assess the viability of GBM cells after OTSSP167 treatment. OTSSP167 imparted a growth-inhibiting effect on various glioblastoma cell lines in a dose-dependent manner. The IC_50_ values of the six cell lines ranged from 100 to 200 nM (**Figure 1A**). We further examined the expression levels of MELK in different GBM cell lines. We found that the levels of MELK in all GBM cell lines had no obvious differences (**Supplementary Files, Figure S1**), which is consistent with the similar sensitivity of OTSSP167 to different cell lines.

To determine the effect of OTSSP167 on GBM proliferation, the U87 and LN229 cells were treated with OTSSP167, followed by an EdU Incorporation assay to assess cell proliferation. Compared with the control group, treatment of U87 and LN229 cells with OTSSP167 resulted in a significant reduction in the rate of cell proliferation (**Figures 1B–E**). The U87 and LN229 cells treated with 200 nM OTSSP167 showed a reduction in the EdU-positive rate to 11.26% and 3.33%, respectively (**Figures 1B, C**). These findings indicate that OTSSP167 significantly inhibits the proliferation of glioblastoma cells in a dose-dependent manner.

To assess the inhibitory effect of OTSSP167 on the long-term growth of GBM, a colony formation assay was performed to detect the effect of OTSSP167 on colony formation in U87 and LN229 cells. Compared with the control group, OTSSP167 treatment reduced the size of colonies in GBM (**Figures 1F–I**). The number of colonies formed by U87 cells treated with 200 nM OTSSP167 decreased to 5.33%, and similar results were observed in LN229 cells. In summary, OTSSP167 significantly inhibited colony formation in GBM.

### OTSSP167 Induces Cell Cycle Arrest in the G2/M Phase

**Figure 2:**
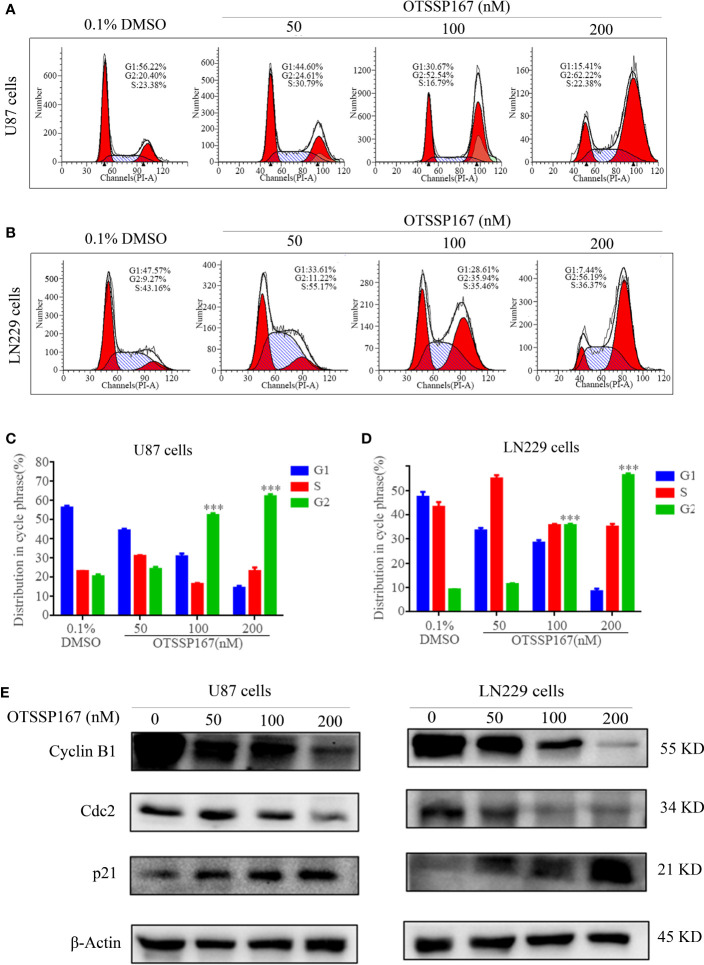
OTSSP167 induces cell cycle arrest at the G2/M phase. **(A, B)** The U87 and LN229 cells were stained with PI after 24 h of OTSSP167 treatment. The results were assessed by flow cytometry. **(C, D)** Quantitative analysis of cell cycle phase distribution of cells in the control and the OTSSP167 treatment groups. **(E)** Western blotting analysis of cell cycle-related protein levels in U87 and LN229 cells treated with OTSSP167 for 24 h with indicated antibodies. All the Data are presented as means ± SEM. ***P<0.001.

To analyze the mechanism by which OTSSP167 inhibits GBM proliferation, flow cytometry was performed to analyze the distribution of cell cycle phases. **Figures 2A–D** show that the U87 and LN229 cells treated with OTSSP167 exhibit a significant increase in the number of cells at the G2/M phase, whereas those at the G1 and S phases decreased. The results indicate that the reduction in cell proliferation induced by OTSSP167 was due to blockage of the cell cycle at the G2/M phase.

To further explore the mechanism by which OTSSP167 regulates the cell cycle, we examined the expression levels of several important regulatory proteins in the G2/M phase. **Figure 2E**, showed that compared with the control group, the expression of the cell cycle inhibitory protein p21 increased with OTSSP167 treatment, whereas that of Cyclin B1 and Cdc2 decreased in a dose-dependent manner. These findings confirm that OTSSP167 induces cell cycle arrest at the G2/M phase by regulating the expression of multiple proteins associated with the G2/M phase.

### OTSSP167 Inhibits GBM Cell Migration and Invasion

**Figure 3:**
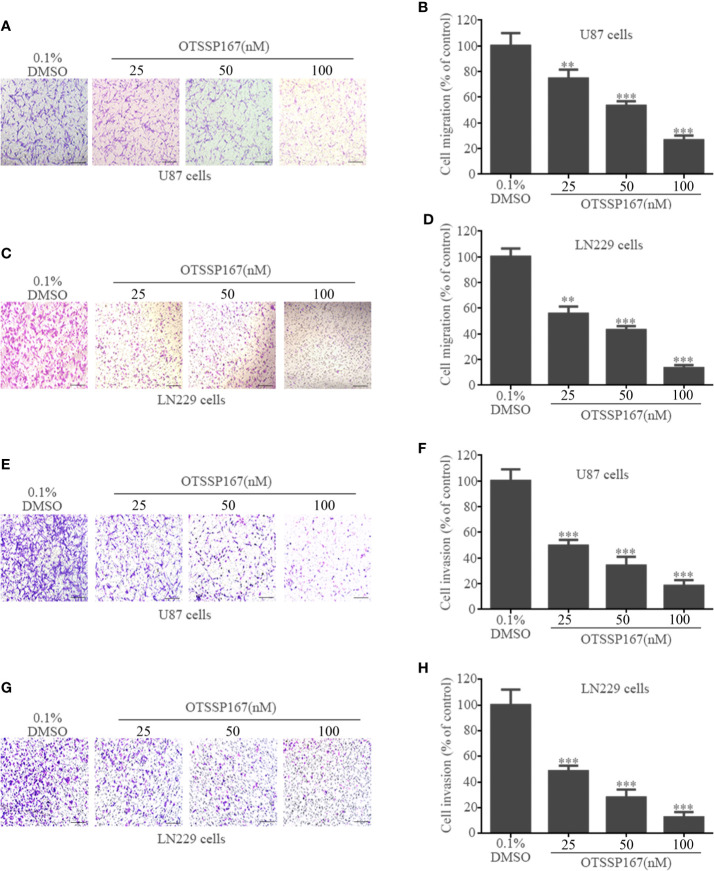
OTSSP167 inhibit GBM cell migration and invasion. U87 and LN229 cells were treated with 0–100 nM OTSSP167 for 24 h, followed by Transwell migration assay (**A–D**, no Matrigel) and invasion assay (**E–H**, addition of Matrigel). All the Data are presented as means ± SEM. **P < 0.01, ***P < 0.001 compared with the 0.1% DMSO treated group.

To clarify the effect of OTSSP167 on GBM cell migration and invasion, Transwell cell migration and invasion assays were performed. Compared with the control group, 50 nM and 100 nM OTSSP167 were used to treat U87 and LN229 cells. The number of migrating U87 cells decreased by 46.67% and 73.34%, respectively (**Figures 3A, B**), whereas that of LN229 cells decreased by 56.72 and 86.37%, respectively (**Figures 3C, D**). Compared with the control group, the U87 and LN229 cells treated with 100 nM OTSSP167 respectively exhibited 81.67% and 87.34% reduction in invasion rates (**Figures 3E–H**). In summary, these data indicate that OTSSP167 significantly inhibits the migration and invasion of GBM in a dose-dependent manner.

### OTSSP167 Inhibits the Proliferation of GBM Cells by Suppressing AKT Phosphorylation

**Figure 4:**
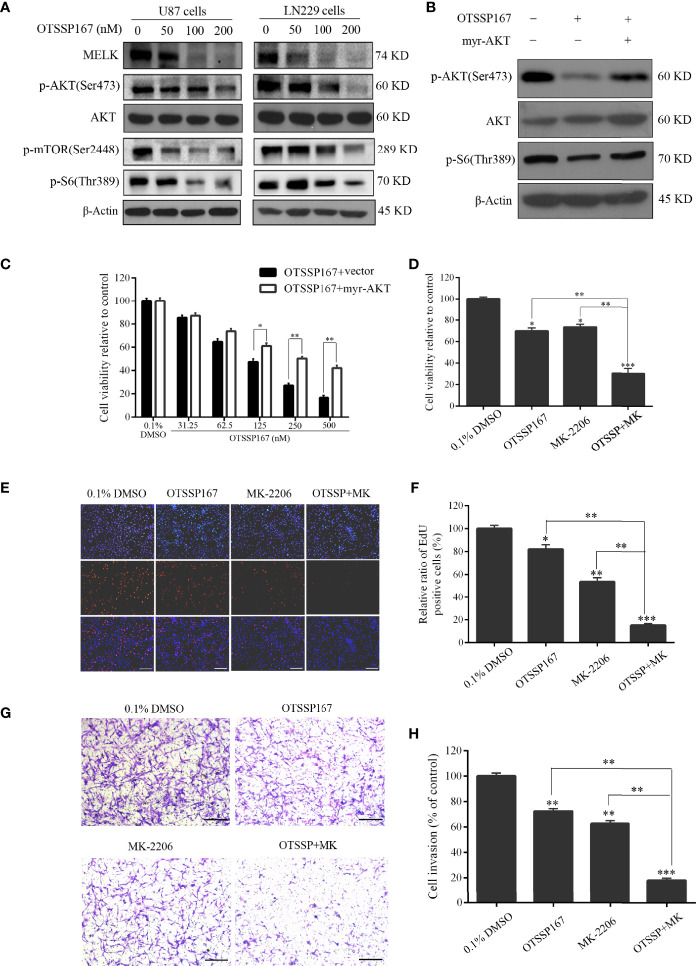
OTSSP167 reduces MELK protein expression and blocks AKT pathway activation, thereby inhibiting the proliferation and invasion of GBM cells. **(A)** U87 and LN229 cells were treated with OTSSP167 for 24 h. Western blotting showing the expression levels of MELK, p-AKT(Ser473), AKT, p-mTOR(Ser2448), and p-S6(Thr389) proteins. **(B)** U87 cells transfected with myr-AKT plasmid were treated with OTSSP167, followed by western blotting to assess changes in p-AKT(Ser473), AKT, and p-S6(Thr389) expression. **(C)** CCK-8 assay shows the effects of OTSSP167 treatment on U87 cells transfected with myr-AKT plasmid compared to the control group. **(D)** CCK-8 assay showing the viability of U87 cells treated with 50 nM OTSSP167 and 1 μM MK-2206 (AKT inhibitor) alone or combined OTSSP167 and MK-2206 treatment for 72 h. **(E)** Measurement of cell proliferation after treating with 50 nM OTSSP167 and 1 μM MK-2206 alone or their combinations by EdU incorporation assay. **(F, H)** Quantitative analysis of proliferative and invading cell numbers. The numbers of proliferative and invading cells were normalized to that of the control group. **(G)** U87 cells were incubated with 50 nM OTSSP167 and 1 μM MK-2206 alone or their combinations. Cell invasive abilities were evaluated by transwell assay. Results were expressed as means ± SEM of three independent experiments. *P < 0.05, **P < 0.01 and ***P < 0.001 compared with control group.

To elucidate the mechanism by which OTSSP167 inhibits the proliferation of GBM cells, western blotting was performed to evaluate the effect of OTSSP167 on the expression of MELK, AKT, and downstream signaling pathway proteins in GBM cells. **Figure 4A** shows that U87 and LN229 cells treated with different concentrations of OTSSP167 showed a decrease in MELK protein level, no change in total AKT protein level, and gradual decrease in phosphorylated AKT level. In addition, OTSSP167 reduced the phosphorylation levels of mTOR and S6, which downstream of the AKT pathway. To test whether OTSSP167 inhibition of GBM proliferation relies on the blockage of the AKT pathway, the AKT consistent activation plasmid myr-AKT was transfected into U87 cells. The cells transfected with myr-AKT expressed rescue p-AKT(Ser473) and p-S6(Thr389) expression when treating with OTSSP167 (**Figure 4B**). The CCK-8 assay revealed that the transfection of the myr-AKT plasmid partially reduced the inhibitory effect of OTSSP167 on GBM cell growth (**Figure 4C**). These findings indicated that OTSSP167 targets the degradation of MELK protein, thereby blocking the AKT pathway and ultimately inhibiting GBM cell proliferation.

We further analyzed whether the combined inhibition of AKT enhances the inhibitory effect of OTSSP167 on GBM cell function. Cells were simultaneously treated with the AKT inhibitor MK-2206 and OTSSP167, resulting in a significant reduction in U87 cell survival compared to the single-drug treatment group (**Figure 4D**). The EdU experiment showed that OTSSP167 and MK-2206 alone inhibit the proliferation of GBM cells. Compared with the single-drug treatment, the combination of the two drugs resulted in a significant reduction in the proportion of EdU-positive cells (**Figures 4E, F**). The Transwell invasion assay showed that MK-2206 enhances the inhibitory effect of OTSSP167 on GBM cell invasion (**Figures 4G, H**). The above data indicate that the combined blockage of the AKT pathway enhances the inhibitory effect of OTSSP167 on GBM proliferation.

### OTSSP167 Significantly Inhibits the Proliferation of GSCs and Neurosphere Formation

**Figure 5:**
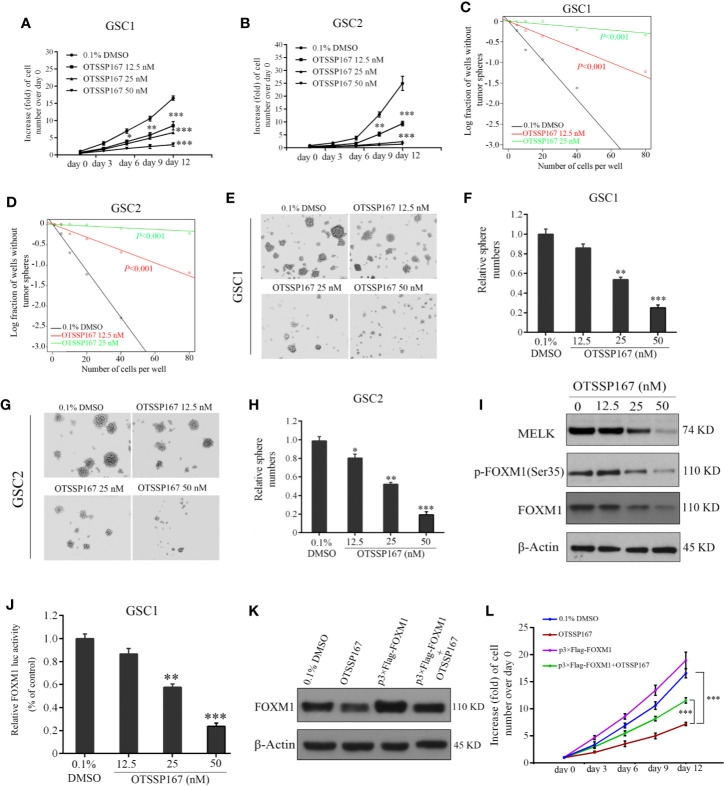
OTSSP167 decreases GSCs proliferation and neurosphere formation by inhibiting the phosphorylation and transcriptional activation of FOXM1. **(A, B)** Effects of OTSSP167 on cell viability in GSC1 **(A)** and GSC2 **(B)**. Cells treated with OTSSP167 were plated in triplicate wells in stem cell media, and then cell viabilities were assessed at the indicated times (day 0–day 12). *In vitro* limiting dilution assays of GSC1 **(C)** and GSC2 **(D)** treated with indicated does of OTSSP167 or 0.1% DMSO. **(E–H)** Effects of OTSSP167 on GSCs neurosphere formation. Representative images of GSC1 **(E)** and GSC2 **(G)** neurosphere and the quantification of sphere numbers of GSC1 **(F)** and GSC2 **(H)** treated with indicated doses of OTSSP167 or DMSO. **(I)** Immunoblot analysis of MELK, p-FOXM1(Ser35), FOXM1 and β-actin in GSC1 treated with indicated doses of OTSSP167. **(J)** OTSSP167 inhibits the transactivation ability of FOXM1. Relative FOXM1 luciferase activity normalized with respect to corresponding renilla luciferase activity is shown. **(K)** The expression levels of FOXM1 were detected by western blot to check the FOXM1 cDNA plasmid transfection efficacy. **(L)** *In vitro* cell viability assay of FOXM1 overexpression in GSC1 cells treated with 25 nM OTSSP167 or the vehicle control. All the Data are presented as means ± SEM. *P < 0.05, **P < 0.01, ***P < 0.001 compared with the 0.1% DMSO treated group.

MELK protein expression is essential to the maintenance of GSC stemness (21). To determine the effect of OTSSP167 on the activity and survival of GSCs, GSC1 and GSC2 cells were treated with OTSSP167, followed by an assessment of their viability. Compared with the control group, the proliferation rates of GSC1 and GSC2 cells treated with OTSSP167 significantly decreased in a dose-dependent manner (**Figures 5A, B**). Subsequently, *in vitro* limiting dilution assay and neurosphere formation assay were used to assess the self-renewal capacity and tumorsphere formation rates of GSCs. We found that OTSSP167 treatment suppressed GSCs self-renewal potential (**Figures 5C, D**). Furthermore, GSC1 and GSC2 neurosphere information gradually decreased with increasing OTSSP167 concentrations (**Figures 5E–H**). Compared with the control group, treatment of GSC1 and GSC2 cells with 25 nM OTSSP167 respectively resulted in a 46.25 and 58.24% decrease in the rate of neurosphere formation. Using 50 nM OTSSP167, the neurosphere formation rate of the GSC1 and GSC2 cells was 15% relative to the control group. These findings showed that OTSSP167 inhibits the maintenance of GSC stemness. More importantly, we found that OTSSP167 are more sensitive in GSCs as compared to the long-term cell lines such as U87 and LN229.

FOXM1 is an important molecule that regulates the maintenance of GSC stemness (32). Western blotting was performed to assess the effect of OTSSP167 on MELK, FOXM1, and p-FOXM1(Ser35) protein expression in GSCs. Our results show that OTSSP167 treatment reduces MELK, FOXM1, and p-FOXM1(Ser35) protein level in GSCs in a dose-dependent manner (**Figure 5I**). The dual luciferase assay revealed a gradual decrease in FOXM1 transcriptional activity with increasing OTSSP167 concentrations (**Figure 5J**). To further confirm the role of FOXM1 in OTSSP167-induced the inhibition in cell viability, we transfected the FOXM1 overexpression plasmid into GSC1 cells, and then we performed the cell viability assay. The expression of Flag-tagged FOXM1 in GSC1 was confirmed by western blot analyses (**Figure 5K**). Overexpression of FOXM1 largely rescued the cell viability of GSC1 after OTSSP167 treatment (**Figure 5L**). Collectively, these results reveal that OTSSP167 reduces the expression of FOXM1, inhibits the transcription activity of FOXM1, and disrupts GSC stemness.

### OTSSP167 Inhibits the Growth of GBM Xenografts *In Vivo*

**Figure 6:**
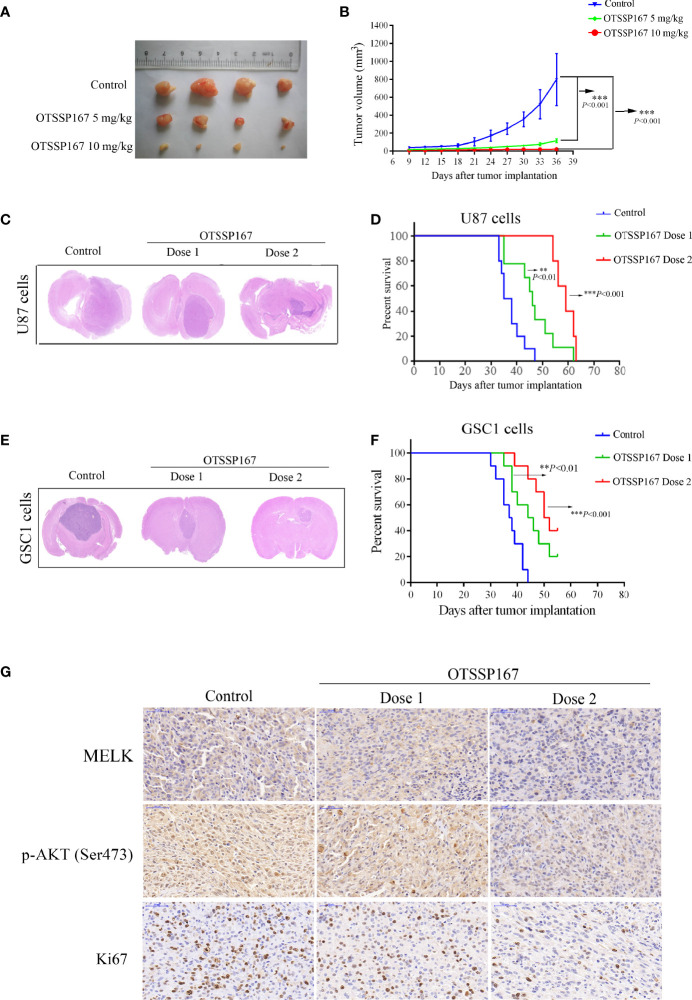
OTSSP167 suppresses tumor growth *in vivo* and increases the survival of animals bearing intracranial GBM. **(A)** Representative tumors isolated from the control and OTSSP167-treated groups of subcutaneous tumor model. **(B)** The mean tumor volumes were assessed at the indicated numbers of days after tumor implantation. **(C)** Mice bearing U87 xenograft tumor were treated with OTSSP167 (5 μL of 1 μM (dose 1) or 2 μM (dose 2) OTSSP167 in 1% DMSO in PBS per mouse) or vehicle control by intratumoral injection once a week for 4 weeks. Representative images of H&E staining of whole brain sections from control group and OTSSP167 treatment group. **(D)** Kaplan-Meier survival curves of mice implanted with U87 cells (n=10, **P < 0.01, ***P < 0.001). *In vivo* animal studies to investigate the effect of OTSSP167 administration on the growth of GSC-driven tumor. Tumor size **(E)** and survival time **(F)** were analyzed by using the above same treatment. The survival time of tumor-bearing mice was counted by the end of the 55th day after tumor implantation. **(G)** Representative IHC staining images of p-AKT(Ser473) and Ki67 expression in U87 xenograft tumor of control and OTSSP167 treatment groups. Sections were counterstained with hematoxylin.

To determine the role of OTSSP167 in inhibiting the proliferation of GBM *in vivo*, a subcutaneous GBM tumor-bearing model using nude mice was constructed for the analysis of the size of the xenograft tumors after OTSSP167 treatment. **Figures 6A, B** show that OTSSP167 treatment significantly inhibits the growth of subcutaneous tumor cells in nude mice. In addition, the tumor volume of the treatment group was significantly smaller than the control.

An orthotopic GBM tumor model using nude mice was subsequently constructed, followed by administration of OTSSP167 to mice. HE staining of tumor tissues showed that the tumors of mice in the treatment group were significantly smaller than the control group (**Figure 6C**). In addition, the tumor-bearing mice treated with OTSSP167 showed significantly prolonged survival. Compared with the control group, the median survival time of the mice of the low-concentration OTSSP167 treatment group was extended by 9 days, and the median survival time of the mice of the high-concentration OTSSP167 treatment group was extended by 22 days (**Figure 6D**, **Supplementary File Table S1**). Moreover, we used GSC1-driven tumor model to further confirm the effects of OTSSP167 on GBM cell growth *in vivo*. We found that OTSSP167 administration markedly inhibited GSC-driven tumor growth (**Figure 6E**) and conferred a significant survival benefit relative to the vehicle control (**Figure 6F**, **Supplementary File Table S2**). These findings indicate that OTSSP167 treatment significantly slowed the growth of GBM tumor in mice and prolonged the survival of the tumor-bearing mice.

To further analyze the effect of OTSSP167 on the proliferation of GBM cells *in vivo* and the expression levels of MELK and p-AKT(Ser473) *in vivo*, IHC was performed on GSC1 tumor tissues to detect the expression of MELK, p-AKT(Ser473) and Ki67. Compared with the control group, the OTSSP167 treatment group showed a significantly lower number of Ki67-positive cells and downregulated MELK and p-AKT(Ser473) expression, which coincided with the results of the *in vitro* experiments (**Figure 6G**), indicating that OTSSP167 inhibits the proliferation of GBM cells *in vivo* by inhibiting the expression of MELK and the phosphorylation of AKT.

## Discussion

GBM is the most common malignant and fatal brain tumors. However, there is still a lack of effective treatment measures. This study showed that OTSSP167 induces cell cycle arrest at the G2/M phase, inhibiting the cell proliferation. In addition, OTSSP167 inhibits GSC cell proliferation and neurosphere formation. *In vivo* experiments further confirmed that OTSSP167 inhibits the growth of GBM and GSC in mice and extends the survival of tumor-bearing mice. More importantly, OTSSP167 inhibits cell growth and invasion by inhibiting the activation of the AKT pathway.

MELK imparts carcinogenic effects on various tumors, including GBM (24, 33). Compared with normal brain tissues, MELK mRNA expression is upregulated in glioblastoma and this expression gradually increases with greater tumor grades (6). Downregulation of MELK expression by siRNA significantly inhibits the anti-apoptotic effect and colony formation in GBM cells (34). Thus, targeting MELK may increase the effectiveness of GBM treatment. This study showed that the MELK inhibitor OTSSP167 blocks the proliferation and cell cycle progression of GBM cell lines, suggesting that it may be potentially utilized in the treatment of GBM. OTSSP167 has a significant inhibitory effect on tumor growth in mice in a dose-dependent manner. Our results show that OTSSP167 treatment reduces the expression of MELK in cell lines and mice. More and more studies have indicated that MELK expression is higher in numerous cancer cells and MELK can drive the most tumor development. However, it has been reported that MELK is not required in triple-negative breast cancer (TNBC) and other cancer types. When knocking out MELK expression, it has no effect on TNBC cell proliferation. Therefore, controversy exists regarding the specific effect of the MELK inhibitor and the role of MELK in cancer. In spite of this, we found that MELK inhibition could suppress GBM cell growth *in vitro* and *in vivo* in our study. Therefore, MELK may be considered as a potential target for the treatment of GBM.

Numerous studies have shown that the frequency of gene mutations and copy number amplification of various regulators in the receptor tyrosine kinase (RTK)/AKT pathway, which is an important regulatory pathway closely related to the pathogenesis and progression of GBM, in glioblastoma is 88% (35, 36). The Akt pathway is usually abnormally activated during the development of GBM (37). Inhibition of AKT activation is a beneficial strategy for tumor treatment, including GBM. This study shows that OTSSP167 treatment inhibits AKT phosphorylation, while further inhibiting the phosphorylation of S6 and mTOR downstream molecules of the AKT pathway, but had no effect on total AKT protein expression. Transfection of the continuously activated AKT in GBM cells partially reversed the inhibitory activity of OTSSP167 on GBM. These results indicate that AKT may be one of the direct or indirect target proteins of MELK in GBM, and the inhibitory effect of OTSSP167 on GBM proliferation is dependent on blocking the AKT pathway.

The presence of stem cells is an important factor in the recurrence of tumor cells and drug resistance (7). The expression of MELK as a stemcell marker molecule was significantly increased in GSCs (6). Downregulation of MELK expression inhibited the survival of GSCs and induced apoptosis of GSCs but had no detectable inhibitory effect on NPCs, indicating that targeting MELK may be an effective strategy to eliminate GSCs (6, 19). This study shows that MELK inhibition by OTSSP167 treatment significantly suppresses the proliferation and neurosphere formation in GSCs. Because GSCs are not sensitive to drugs, the development of drug resistance and relapse may be important factors in the pathogenesis of GBM. Notably, GSCs were particularly sensitive to the MELK inhibitor OTSSP167. Moreover, the inhibitory effect of OTSSP167 on the proliferation of GSCs was four-fold more effective than GBM cells. Recently, several research reports have shown that OTSSP167 can have off-target effects on Aurora B kinase (38, 39). However, OTSSP167 inhibited Aurora B kinase activity with IC50 approximately at ~25 nM (38). The inhibitory activity of OTSSP167 to MELK is many times more than that to Aurora B kinase. In addition, OTSSP167 has little effect on cancer cells with low MELK expression (3, 40) or normal cells (4, 5, 41). MELK as an important cancer stem cell marker was highly expressed in GSCs (6, 19), which may be one of the reasons why GSCs is more sensitive to OTSSP167.

FOXM1 is an important regulatory factor for the maintenance of GSC stemness and is one of the substrates of MELK (6). This study showed that OTSSP167 treatment reduced the expression of MELK in GSCs and reduced the expression of total FOXM1 and p-FOXM1(Ser35). As the concentration of OTSSP167 increased, the transcriptional activity of FOXM1 gradually decreased. Notably, the declined cell proliferation of GSC1 by OTSSP167 treatment were implicated to be rescued in part by FOXM1 overexpression, indicating that OTSSP167 inhibits the phosphorylation and transcription-activation abilities of FOXM1 by reducing MELK expression, thereby inhibiting the survival and maintenance of GSC stemness. These findings indicate that targeted inhibition of MELK may be an effective strategy for eliminating GSCs.

The blood–brain barrier (BBB) is considered as the bottleneck in brain drug development and is an important consideration in determining whether a drug can penetrate the brain during treatment of glioblastoma (42). Multidrug resistance protein 1 (MDR1) and breast cancer resistance protein (BCRP) are two important efflux drug transporters that are highly expressed at the BBB (43). These can actively expel these substrates from the brain, limiting the penetration of compounds in the brain (43). A recent study has shown that OTSSP167 is a substrate of MDR1 and BCPR. Under physiological conditions, OTSSP167 mainly exists in the hydrophilic form, which limits the ability of OTSSP167 to penetrate the BBB (44). Their results has shown that simultaneously knocking out *MDR1* and *BCPR* significantly increases OTSSP167 levels in the brain, indicating that OTSSP167 fails to penetrate the BBB, and the effective concentration in the brain has yet to be established (44). This study investigated whether OTSSP167 significantly inhibits the growth of intracranial tumors in mice. Considering the limited BBB permeability of OTSSP167, we conducted intracranial injection for *in vivo* studies. Compared with the control group, the survival time of mice in the high-concentration OTSSP167 treatment group was extended by 22 days. Further research strategies for targeting MELK to treat glioblastoma should be focused on the following two aspects (1): development of MELK inhibitors with better permeability in the brain, and (2) using a nano-drug delivery system for direct transport of OTSSP167 into the brain to increase its concentration in the brain.

## Conclusions

In summary, our results demonstrate that MELK inhibitor OTSSP167 significantly inhibits the proliferation of GBM by directly or indirectly blocking the AKT pathway. Furthermore, OTSSP167 also reduces the self-renewal capacity of GSCs by inhibiting FOXM1 phosphorylation and transcription activity. Interestingly, the inhibitory effect of OTSSP167 on the proliferation of GSCs was more effective than GBM cells. OTSSP167 simultaneously inhibits the proliferation of GBM and the self-renewal capacity of GSCs, indicating that MELK may be a potential and promising therapeutic target for GBM treatment.

## Data Availability Statement

The original contributions presented in the study are included in the article/**Supplementary Material**. Further inquiries can be directed to the corresponding authors.

## Ethics Statement

The animal study was reviewed and approved by Xuzhou Medical University.

## Author Contributions

XL and RY designed this study. XZ and JW performed the main experimental procedures *in vitro*. YW conducted the animal assays *in vivo*. GL, HL, JY, and RW carried out partial experiments. JL performed the statistical analysis. XL wrote this manuscript. All authors contributed to the article and approved the submitted version.

## Funding

The research was supported by National Natural Science Foundation of China (No. 81772658, 81972345, 81670142); Jiangsu Provincial Key Research and Development Program (BE2017636, BE2017638); Natural Science Foundation of Jiangsu Province (BK20180104); Jiangsu Qing Lan Project for XL.

## Conflict of Interest

The authors declare that the research was conducted in the absence of any commercial or financial relationships that could be construed as a potential conflict of interest.

## Abbreviations

GBM, Glioblastoma multiforme; GSCs, Glioblastoma stem-like cells; MELK, Maternal embryonic leucine zipper kinase gene; FOXM1, Forkhead box M1; CCK-8, Cell Counting Kit-8; EdU, 5-ethynyl-2’-deoxyuridine.
