# Corrigendum: MELK Inhibition Effectively Suppresses Growth of Glioblastoma and Cancer Stem-Like Cells by Blocking AKT and FOXM1 Pathways

**DOI:** 10.3389/fonc.2022.911817

**Published:** 2022-04-22

**Authors:** Xu Zhang, Jie Wang, Yifeng Wang, Guanzheng Liu, Huan Li, Jiefeng Yu, Runqiu Wu, Jun Liang, Rutong Yu, Xuejiao Liu

**Affiliations:** ^1^ Institute of Nervous System Diseases, Xuzhou Medical University, Xuzhou, China; ^2^ Department of Neurosurgery, Affiliated Hospital of Xuzhou Medical University, Xuzhou, China; ^3^ The Graduate School, Nanjing Medical University, Nanjing, China; ^4^ Department of Neurosurgery, The Second Affiliated Hospital of Xuzhou Medical University, Xuzhou, China

**Keywords:** glioblastoma multiforme, glioblastoma stem-like cells, maternal embryonic leucine-zipper kinase, OTSSP167, targeted therapy

In the original article, there was a mistake in **Figures 1D**, **4G**, **6E** as published. Incorrect images were used in the figure assembly process. The corrected **Figures 1**, **4** and **6** appear below.

**Figure 1:**
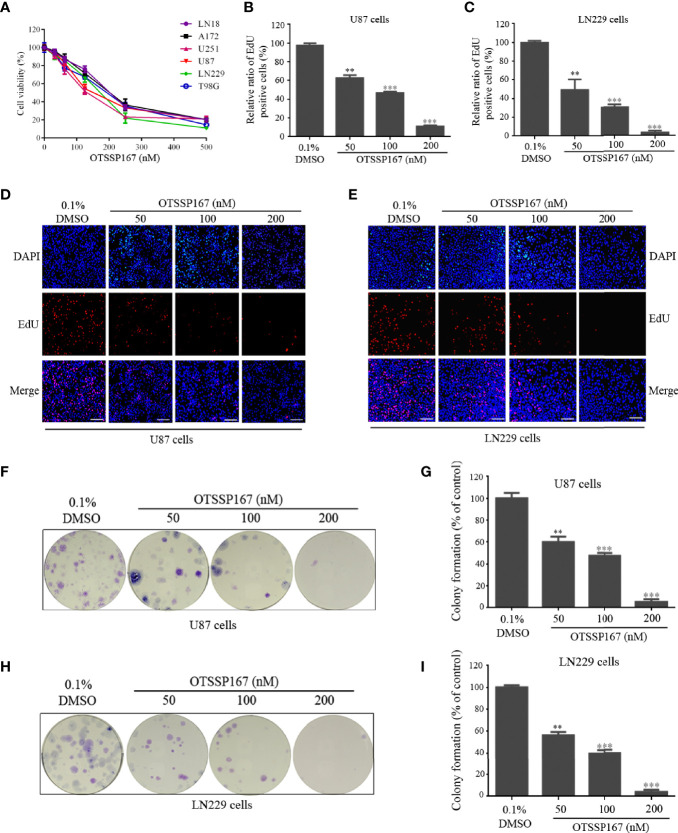
OTSSP167 inhibits GBM cell proliferation and colony formation. **(A)** CCK-8 viability analysis of cells treated with six OTSSP167 concentrations, including 0 nM, 31.25 nM, 62.5 nM, 125 nM, 250 nM, and 500 nM for 72 h. **(B–E)** The U87 and LN229 cells were treated with indicated concentrations of OTSSP167 for 24 h, and the EdU assay was performed to assess cell proliferation. Panels **(B, C)** show the results of the quantitative analysis of the EdU test; panels **(D, E)** show the representative images of EdU analysis after OTSSP167 treatment of the U87 and LN229 cells. **(F–I)** OTSSP167 inhibits colony formation in U87 and LN229 cells in a dose-dependent manner. Quantitative analysis of the results of the colony formation experiment was performed. All the Data are presented as means ± SEM. **P < 0.01, ***P < 0.001 compared with the 0.1% DMSO treated group.

**Figure 4:**
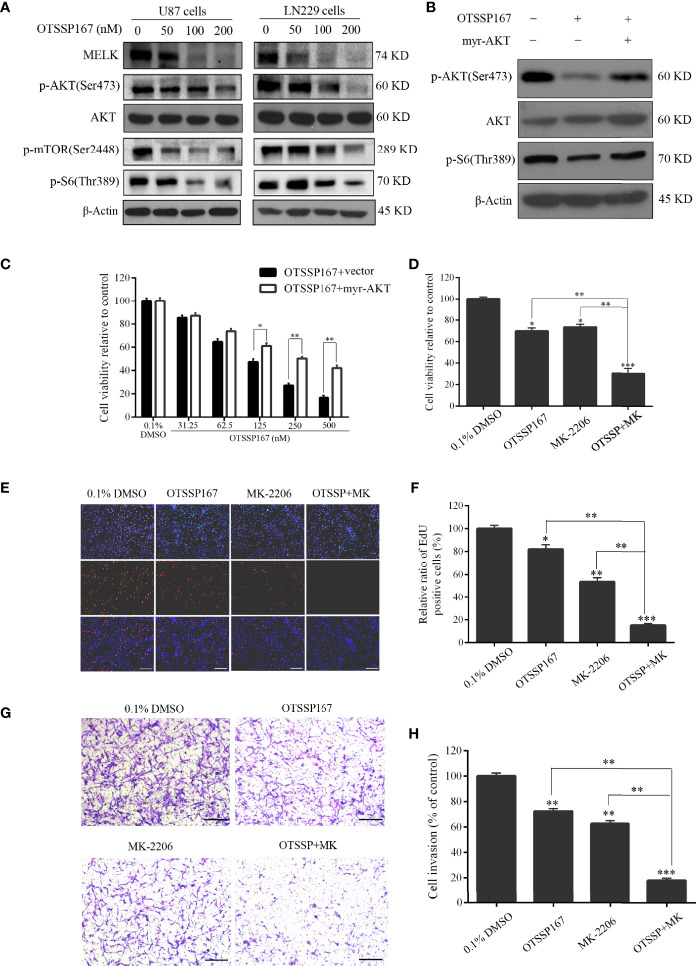
OTSSP167 reduces MELK protein expression and blocks AKT pathway activation, thereby inhibiting the proliferation and invasion of GBM cells. **(A)** U87 and LN229 cells were treated with OTSSP167 for 24 h. Western blotting showing the expression levels of MELK, p-AKT(Ser473), AKT, p-mTOR(Ser2448), and p-S6(Thr389) proteins. **(B)** U87 cells transfected with myr-AKT plasmid were treated with OTSSP167, followed by western blotting to assess changes in p-AKT(Ser473), AKT, and p-S6(Thr389) expression. **(C)** CCK-8 assay shows the effects of OTSSP167 treatment on U87 cells transfected with myr-AKT plasmid compared to the control group. **(D)** CCK-8 assay showing the viability of U87 cells treated with 50 nM OTSSP167 and 1 μM MK-2206 (AKT inhibitor) alone or combined OTSSP167 and MK-2206 treatment for 72 h. **(E)** Measurement of cell proliferation after treating with 50 nM OTSSP167 and 1 μM MK-2206 alone or their combinations by EdU incorporation assay. **(F, H)** Quantitative analysis of proliferative and invading cell numbers. The numbers of proliferative and invading cells were normalized to that of the control group. **(G)** U87 cells were incubated with 50 nM OTSSP167 and 1 μM MK-2206 alone or their combinations. Cell invasive abilities were evaluated by transwell assay. Results were expressed as means ± SEM of three independent experiments. *P < 0.05, **P < 0.01 and ***P < 0.001 compared with control group.

**Figure 6:**
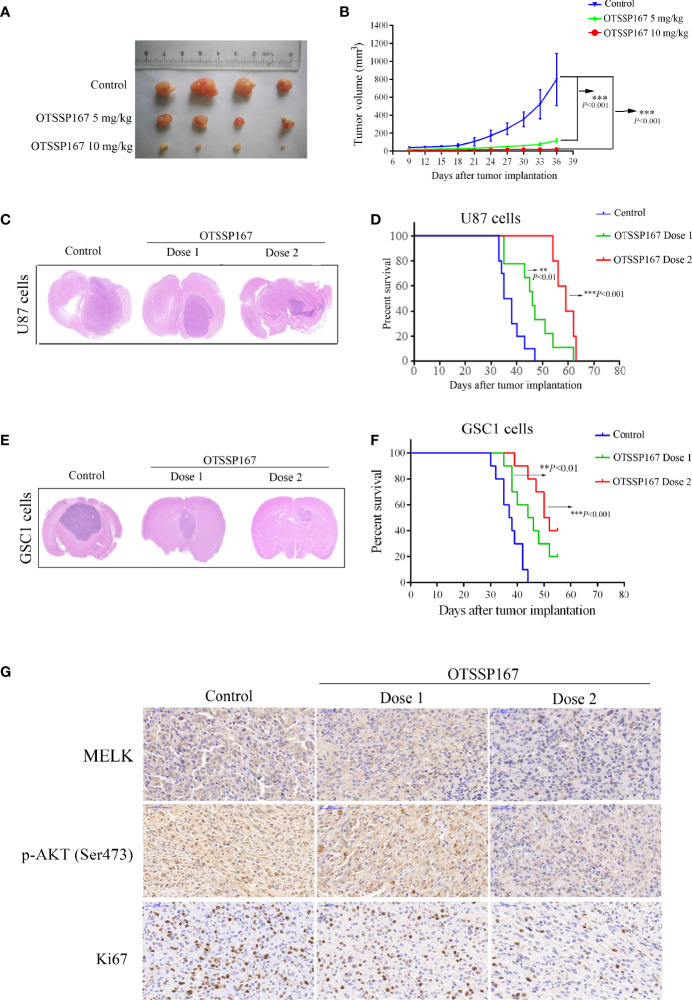
OTSSP167 suppresses tumor growth *in vivo* and increases the survival of animals bearing intracranial GBM. **(A)** Representative tumors isolated from the control and OTSSP167-treated groups of subcutaneous tumor model. **(B)** The mean tumor volumes were assessed at the indicated numbers of days after tumor implantation. **(C)** Mice bearing U87 xenograft tumor were treated with OTSSP167 (5 μL of 1 μM (dose 1) or 2 μM (dose 2) OTSSP167 in 1% DMSO in PBS per mouse) or vehicle control by intratumoral injection once a week for 4 weeks. Representative images of H&E staining of whole brain sections from control group and OTSSP167 treatment group. **(D)** Kaplan-Meier survival curves of mice implanted with U87 cells (n=10, **P < 0.01, ***P < 0.001). *In vivo* animal studies to investigate the effect of OTSSP167 administration on the growth of GSC-driven tumor. Tumor size **(E)** and survival time **(F)** were analyzed by using the above same treatment. The survival time of tumor-bearing mice was counted by the end of the 55th day after tumor implantation. **(G)** Representative IHC staining images of p-AKT(Ser473) and Ki67 expression in U87 xenograft tumor of control and OTSSP167 treatment groups. Sections were counterstained with hematoxylin.

The authors apologize for this error and state that this does not change the scientific conclusions of the article in any way. The original article has been updated.

## Publisher’s Note

All claims expressed in this article are solely those of the authors and do not necessarily represent those of their affiliated organizations, or those of the publisher, the editors and the reviewers. Any product that may be evaluated in this article, or claim that may be made by its manufacturer, is not guaranteed or endorsed by the publisher.

