# Supplementary material for: MELK Inhibition Effectively Suppresses Growth of Glioblastoma and Cancer Stem-Like Cells by Blocking AKT and FOXM1 Pathways
Source: Front Oncol. 2021 Jan 14;10:608082. doi: 10.3389/fonc.2020.608082 (PMC7842085; doi:10.3389/fonc.2020.608082)
Supplement: Supplementary file 1 [file DataSheet_1.pdf]

Supplementary file

Table S1. The data of survival studies for the nude mice bearing U87 tumor cells

Days	Group A	Group B	Group C
33	1		
33	1		
34	1		
35	1		
35	1		
38	1		
38	1		
40	1		
43	1		
47	1		
35		1	
35		1	
43		1	
45		1	
46		1	
47		1	
51		1	
54		1	
62		1	
54			1
54			1
56			1
56			1
59			1
59			1
62			1
62			1
63			1

Days: The died days of the nude mice after tumor implantation

Group A: Vehicle control

Group B: OTSSP167 Dose 1 (5 μL of 1 μM OTSSP167 in 1% DMSO in PBS per mouse)

Group C: OTSSP167 Dose 2 (5 μL of 2 μM OTSSP167 in 1% DMSO in PBS per mouse)

1 represents the number of died mouse

Table S2. The data of survival studies for the nude mice bearing GSC1 tumor cells

Days	Group A	Group B	Group C
30	1		
32	1		
35	1		
35	1		
37	1		
38	1		
39	1		
42	1		
42	1		
44	1		
35		1	
38		1	
38		1	
40		1	
44		1	
46		1	
48		1	
52		0	
55		0	
39			1
44			1
47			1
50			1
50			1
52			0
55			0
55			0
55			0

Days: The died days of the nude mice after tumor implantation

Group A: Vehicle control

Group B: OTSSP167 Dose 1 (5 μL of 1 μM OTSSP167 in 1% DMSO in PBS per mouse)

Group C: OTSSP167 Dose 2 (5 μL of 2 μM OTSSP167 in 1% DMSO in PBS per mouse)

1 represents the number of died mouse

0 means the mouse is not dead

All the mice were treated with OTSSP167or vehicle control by intratumoral injection once a week for 4 weeks.

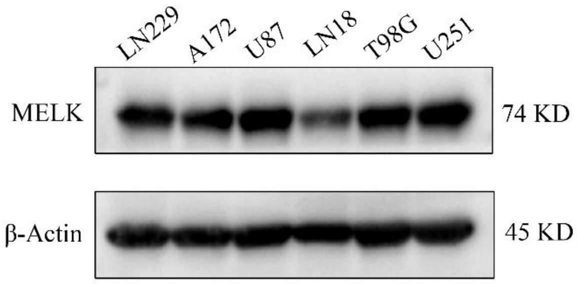

Figure S1. Expression levels of MELK in different GBM cell lines. Immunoblot analysis of MELK in LN229, A172, U87, LN18, T98G and U251 cells. β-Actin as a loading control.
